# A Unique Glassy Cell Carcinoma (GCC) of the Cervix Diagnosed during Pregnancy—A Case Report

**DOI:** 10.3390/healthcare10081583

**Published:** 2022-08-21

**Authors:** Marlena Grabowska, Ewa Baum, Małgorzata Lewandowska, Stefan Sajdak, Klaudia Dolińska-Kaczmarek, Monika Englert-Golon

**Affiliations:** 1 Department of Gynaecology Obstetrics and Gynaecological Oncology, Division of Gynecological Surgery, Poznan University of Medical Sciences, 60-535 Poznan, Poland; 2 Department of Social Sciences and the Humanities, Poznan University of Medical Sciences, 60-806 Poznan, Poland; 3 Department of Biochemistry and Molecular Biology, Poznan University of Medical Sciences, 60-781 Poznan, Poland

**Keywords:** glassy cell carcinoma, cervix, pregnancy, treatment

## Abstract

Glassy Cell carcinoma (GCC) of the cervix is classified as a unique, aggressive neoplasm, with different sensitivity to chemotherapy and radiotherapy. It is such an extremely rare tumor that it is practically not observed during pregnancy. Information on the coexistence of cervical GCC with pregnancy is also unique, so it seems extremely important to disseminate it in order to develop the most effective treatment regimen. Additionally, making any decisions regarding therapeutic methods during pregnancy encounters great ethical problems. We present the case of a 26-year-old pregnant woman, 18 weeks gestation, diagnosed with GCC of the cervix, IB3 grade in the International Federation of Gynecology and Obstetrics (FIGO) scale. Despite the unfavorable prognosis, the use of chemotherapy in a pregnant patient brought on a favorable therapeutic effect, without any negative effects on the fetus. The article also presents a literature review on the epidemiology, pathology, immunohistochemistry, treatment and prognosis of this rare disease.

## 1. Introduction

Glassy cell carcinoma (GCC) of the cervix is classified as a unique, aggressive neoplasm, with different sensitivity to chemotherapy and radiotherapy. It accounts for 1–5% of all cervical cancers in non-pregnant women. It is such an extremely rare tumor that it is practically not observed during pregnancy. Information on the coexistence of cervical GCC with pregnancy is also unique, and in the literature, there is only one case of a pregnant patient with GCC [1]. Making any decisions regarding therapeutic methods during pregnancy encounters great ethical problems. It is always accompanied by concerns about the impact of the applied therapy on the developing fetus. From a legal point of view, taking any action or not taking any action requires the patient’s written informed consent each time.

GCC of the cervix is an undifferentiated neoplasm with features of adenocarcinoma and squamous cell carcinoma. Cherry and Gluksman described this neoplasm as an undifferentiated glandular-squamous neoplasm of the cervix with an aggressive course, poorly responding to radiotherapy. The degree of malignancy of cancer depends on the area of undifferentiated structures present in the tumor. In the analysis of several cases of non-pregnant patients with this cancer, rapid progression and distant metastases were confirmed [2,3]. GCC of the cervix is made up of large cells with clear cell boundaries and a fine-grained cytoplasm with a glassy eye. Extensive leukocyte infiltrates with a predominance of eosinophytes and single plasma cells often occur in the stroma [3,4].

Despite reports in the literature regarding the incidence of cervical GCC in the population of non-pregnant women, there are still no large statistical studies. Zolciak—Siwińska and Jońska—Gmyrek conducted an analysis of the literature describing about 300 cases of GCC in non-pregnant patients since its definition by Gluksman and Cherry until 2014 [5]. It needs to be highlighted that no trials or large studies were identified because of the rarity of GCC. Information on the coexistence of cervical GCC with pregnancy is unique and needs to be disseminated in order to conduct a wider case study. When preservation of the pregnancy is desired, optimal treatment is a major challenge to all [6].

Since the cancer was first described, the prognosis was very poor and the 5-year OS ranges between 13% and 30%; sometimes, OS is less than one year [5]. The turning point seems to be the multimodal therapy, which changed this grim outlook for early stage patients and 5-year survival is now 80% in patients with FIGO stage I [7]. It, therefore, seems extremely important to quickly diagnose what is needed to implement the most optimal multimodal therapy and will benefit overall survival (OS) and progression-free survival (PFS) [8].

We present the case of a pregnant patient with diagnosed GCC of the cervix. We describe symptoms, diagnosis, choice of treatment methods, childbirth and post-treatment oncological care. Perhaps this will contribute to the development of the most effective treatment regimen, which will allow, above all, the treatment of pregnant women with the lowest percentage of complications.

## 2. Case Description

### 2.1. Diagnosis

A 26-year-old patient in her first pregnancy, at 18 weeks of gestation, was admitted to the hospital due to vaginal bleeding. Before admission to hospital, the pregnancy was normal without any disturbing symptoms.

The speculum examination revealed the presence of a bloody vaginal discharge and the entire cervix was turned into a tumor with a diameter of about 60 mm. Transvaginal ultrasound examination revealed a hyperechoic space 70 × 36 mm in the cervix from the vaginal side. Six months before pregnancy, no pathological changes were found in cytology. Due to spotting from the genital tract, which appeared in the first trimester of pregnancy, a control cytology was performed at the beginning of the second trimester and high-grade intraepithelial lesions (HSIL) were diagnosed. Cytological examination was extended to include genotyping of human papillomavirus (HPV), confirming the presence of high-risk human papillomavirus type 16 (HR HPV 16).

In the light of the above information, taking into account the presence of macroscopic cervical lesions, it was decided to perform a direct cervical biopsy. Four slices of the cervical lesion were taken. Histopathological examination extended with immunohistochemical diagnostics, confirming the presence of GCC of the cervix.

In the microscopic image, nests of low-differentiated cancer with a large atypia and a scanty stroma with an inflammatory reaction with features of glandular and squamous cell carcinoma were visible. Immunohistochemical reactions were positive for p63 and CKEAE1/AE3, focal for Vimentin and progesterone receptor (PR). However, there was no expression of the estrogenic receptor (ER). The reaction with the proliferative antigen Ki67 showed about 50% of the nuclei of cancer cells, while the histochemical reaction to mucus with mucicarmine was shown in the cytoplasm of a few cells.

Magnetic Resonance Imaging (MRI) of the abdominal cavity and pelvis during pregnancy did not reveal infiltrative changes and metastatic foci.

### 2.2. Therapeutic Decisions

Due to the very rare type of malignant tumor and the coexistence of early pregnancy, the council presented the patient and her family with the following therapeutic options:

- Termination of pregnancy with elective surgery for radical removal of the uterus, followed by oncological treatment.
- Procedure waiting up to 32 weeks pregnancy with pharmacological stimulation of the maturity of the fetal respiratory system, then cesarean section with simultaneous radical removal of the uterus with appendages and further oncological treatment.
- Initiation of treatment during pregnancy with a risk of irreversible damage to the fetus as a result of radio and/or chemotherapy.
- Waiting procedure until the start of uterine contractile function—only by the patient’s decision.

For both the patient and the therapeutic team, the decision to choose treatment was a major moral, ethical and clinical challenge. In current Polish law, the pregnancy posed a threat to the life and health of the pregnant woman. This circumstance is referred to as a health condition under which termination of pregnancy is allowed. The expression “health risk” in the described case is understood as the existence of a disease that will worsen if the pregnancy continues and not only poses a threat to the well-being of the pregnant woman [9]. Medical staff, guided by the principle of the patient’s well-being and the principle of non-harm, offered the woman several options with a view to respecting the right to autonomous decision-making regarding the life of her own patient and her child [10]. Eventually, the patient decided to maintain the pregnancy with the simultaneous inclusion of chemotherapy, thus, giving up the possibility of irradiation treatment during pregnancy. Such a decision, which was a partial abandonment of treatment, constituted the autonomous will of the patient with signs of negative autonomy [11]. It should be noted that the staff was in accordance with the patient’s decision.

### 2.3. Treatment

Oncologists proposed neoadjuvant therapy [TP] Paclitaxel (135.0 mg/m^2^) and Cisplatin (75.0 mg/m^2^), minimum III courses every 3 weeks. Then, at the 28th week of pregnancy, an imaging examination—magnetic resonance imaging—is planned. Treatment proceeded without complications.

After the third course of chemotherapy, the effects of the treatment were evaluated. In the examination in the speculum, the vaginal part of the cervix was slightly enlarged, reddened, the internal outlet closed, normal discharge and bleeding or spotting was not found. The absence of a cervical tumor was observed, which confirmed the effectiveness of chemotherapy and a good decision on the method of treatment. In the ultrasound examination, normal development of the fetus was confirmed. MRI of the abdominal cavity performed in T1 and T2 images, with a DWI sequence, excluded the presence of metastatic foci.

Due to the positive response to the treatment and the absence of obstetric complications, the decision to continue chemotherapy was made after the end of the procedure. After four courses from five-course chemotherapy, the pregnant woman came to the hospital with a ruptured membrane. Antibiotic therapy, stimulation of the fetal lungs, intensive supervision of the patient and fetus were used. At 34 weeks of pregnancy, after 8 days from the fifth course, regular contractile activity of the uterus began, so the decision to have a cesarean section was made. The newborn weighed 1960 g and received the following score according to the Apgar scale of 6, 7, 8 (in the 1st, 3rd and 5th minute, respectively). Due to the use chemotherapy in pregnancy and premature delivery, the newborn underwent transacute head ultrasound, echocardiography and specialist consultations, which showed no abnormalities.

A month after the cesarean section, the patient underwent radical hysterectomy and bilateral salpingo-oophorectomy with bilateral sentinel node biopsy and pelvic node dissection. Eight lymph nodes were obtained. The histopathological result confirmed the presence of a 2.5 cm focal point with scattered neoplastic cells. The presence of angioinvasion was also confirmed. Pelvic lymph nodes were unchanged.

Four weeks after surgery, three courses of chemotherapy were added to TP Paclitaxel (135.0 mg/m^2^) and Cisplatin (75.0 mg/m^2^). After a month, the patient was qualified for teletherapy and brachytherapy. Teleradiotherapy with 6 MV photons was used. The patient received a dose of 50.40 Gy in 28 fractions for the pelvic area. The next day, after the end of teleradiotherapy, the patient was treated with HDR brachytherapy. Three applications of two ovoids of 6 Gy each for reference isodose were performed. She received a total dose of 18 Gy around the top of the vagina. Treatment proceeded without complications.

### 2.4. Oncological Control

Three years have passed since the diagnosis of Cervical GCC in the patient and she is under the control of the Gynecological Oncology Clinic. Visits take place every 6 months. To date, no recurrences have been observed. The patient describes the state of her health as very good. The child is developing properly.

Therefore, it seems correct to say that both the patient and the therapeutic team, in the face of many ethical dilemmas, made the right decision about the choice of treatment method, thus, affecting the life of the mother and child. In the context of changing legal regulations or ethical principles, no position is fully adequate to guide the patient’s decisions. It is up to the woman and/or her partner to decide autonomously about their own life and that of their child.

## 3. Discussion

We presented the symptoms, diagnosis and treatment of the case of the pregnant patient with diagnosed GCC of the cervix. Due to the lack of literature data confirming the coexistence of this cancer with pregnancy, apart from the case described so far [1], non-pregnant patients are the reference point. The rarity of this neoplasm (in non-pregnant women) also influenced the citation of distant literature data.

The most common cervical cancer is squamous cell carcinoma, which accounts for about 80% of all malignant cervical tumors. About 10% are adenocarcinomas. It needs to be highlighted that only 10% of all malignant tumors are other types, such as glandular squamous cell carcinoma, carcinoid and non-epithelial neoplasms [2,3].

According to the data available in the literature, patients with GCC are about 10 years younger than patients with other types of cervical cancer and the case analyzed by us confirms these observations [5,12]. It is highly rare tumor, but some studies have noted an association with pregnancy [13,14]. It is possible that some authors relate this tumor to pregnancy because it appears at a younger age, with a higher probability that the patient is pregnant. 

The first symptom of GCC, similar to other types of cervical cancer, is abnormal vaginal bleeding, which also occurred in our pregnant patient [3,4]. In the study of Wang et al., 100% of patients with Cervical GCC went to the doctor due to vaginal bleeding [15]. According to the International Federation of Gynecologists and Obstetricians, the stage of IB1 is found in 93.3% in non-pregnant patients [3,5,16]. Wang et al., in an analysis of 20 cases of women with cervical GCC, described a diagnosis of stage I cancer in 75% of patients. In the second degree in 20% of patients and in the III degree in 5% of patients [15]. In the case presented in this paper, the level of advancement was defined as IB3.

The therapeutic effect correlates with the stage of the cancer. Kędzia et al. described the case of a 24-year-old patient with cervical GCC in grade IB1 G3, who, after radio and chemotherapy, survived without a recurrence of at least 12 months [3]. Analyzing the results of Wang et al., it turned out that in FIGO I, relapse occurred after 28 months in only one patient where pelvic radiation and combined chemotherapy were used instead of surgery. Local recurrence usually occurs at the apex of the vagina, parametrium and para-aortic lymph nodes. Distant metastases beyond the pelvis minor are also observed, including to the lungs, liver, spleen and bones [5,15]. A good response to adjuvant chemotherapy after surgical treatment is also confirmed by Takahashi’s analyses. In a 44-year-old patient with grade IB1 who did not receive chemotherapy, recurrence appeared less than 12 months after a radical hysterectomy with pelvic and nodes. In contrast, two patients with grade IB2 and IIA who received adjuvant chemotherapy after surgical treatment had a recurrence-free time of 4.5 and 9 years, respectively [17]. Yoon et al. reported an excellent result in three patients with FIGO IIB who had no recurrence or metastasis after chemioradiotherapy for eight years [18]. Takekuma also reports a beneficial therapeutic effect after adjuvant chemotherapy in a patient with grade IB2. Based on their observations, the authors speculate that chemotherapy is an appropriate therapeutic method for patients with GCC [19]. Nasu et al. suggested that the best therapeutic effect assessed on the basis of time free from recurrence is achieved by patients after combination therapy, which includes surgical treatment, chemotherapy and radiotherapy [20]. Piura et al. recommended multimodal treatment for early stage cervical GCC, which includes primary surgery with radical hysterectomy and lymphadenectomy, followed by pelvic radiotherapy with systemic chemotherapy as the most effective [7]. Lotocki et al. described 5-year OS in the early stages after multimodal therapy of over 80% compared to 45% after surgery and 50% after radiotherapy [8]. The great importance of adjuvant treatment: radio-chemotherapy based on paclitaxel-carboplatin after surgery underlines Nasu et al. When the case is high risk for para-aortic lymph node metastasis due to the presence of pelvic lymph node involvement, para-aortic lymphadenectomy below the origin of the inferior mesenteric artery is worth considering [20]. Radical radiotherapy as adjuvant treatment improves OS and PFS in cervical GCC [8,21]. Recurrence rate by treatment was highest among patients treated only with surgery, as compared to patients treated with surgery followed by radiation or radiation only. Randomized studies have demonstrated a reduction in recurrence rate and significant improvement in OS when chemotherapy was used with radiotherapy [22]. Our observations are consistent with these reports. It should be noted that in the described case, radiation therapy was used 7 months after diagnosis because of the pregnancy. Thus, chemotherapy was crucial in the first 6 months of treatment. Undoubtedly, however, it should be emphasized once again that the data cited for comparison from the literature concern patients with GCC of the cervix who are not pregnant, where making a decision about the type of therapy is devoid of ethical dilemmas.

In older studies, the 5-year OS rate was between 13% and 30% [5]. Tsukahara et al. observed that 13 out of 14 patients with GCC of the cervix died within 25 months [23]. Data from a meta-analysis of 292 cases with GCC indicated that OS for all cases was 25.0 months and the 5-year OS was 55.0% Guitarte et al. reported that 5-year OS was lower for GCC, as compared to other cervical cancer (55% versus 75%). The meta-analysis of 148 cervical GCCs in early and advanced stages shows that median OS was 25 months. There was a significant difference in survival by stage but no interaction between race and OS [22]. The cumulative 5-year OS for all stage I GCCs was 48% compared with the stage I nonglassy cell adenocarcinomas 61% [24]. Boustani et al. reported 5-year OS of 85.2% in early stage tumors versus 76.4% for locally advanced cases [12]. The patient in stage IVB, described by Kosińska-Kaczyńska et al., died within six months of the diagnosis [25]. Multimodal therapy changed this grim outlook for early stage patients and 5-year survival is now 80% in patients with FIGO stage I [7]. 

PFS rates at 5 years were 86.4% (CI: 63.4–95.4) for early stage versus 75.9% (CI: 55.2–89.2) for locally advanced stages, respectively. Further, 33% patients experienced a tumor relapse and the median time was 16 months (range: 5 months to 16 years) [12]. In older studies, most recurrences were identified within 24 to 31 months of primary therapy [5]. Zhonghua et al. reported that in five analyzed patients, PFS ranged from 25 days to 33 months [26]. In the case we analyzed, we did not see recurrence for 38 months, since the diagnosis was made, which is confirmed by imaging examinations.

A significant association was found between cervical glassy cell carcinoma and HR HPV infection. HPV 18, 16 and 32 were detected in 67% of subjects [11]. In the case presented in our work, a positive HPV 16 DNA result was obtained, as in one of the four cases described by Kim et al. of patients with GCC of the cervix [27]. The correlation of cervical GCC with a positive HPV result is also confirmed by the analysis of five cases of cervical GCC by Jung et al. It should be noted, however, that more often, in three patients, it was type 18, while type 16 and 31 occurred independently in the other two patients [2]. The available data also highlight the possibility of co-occurrence of both non-oncogenic and oncogenic types in individual patients [27].

The use of chemotherapy treatment in the second and third trimesters of pregnancy does not pose a threat to the fetus [4,6]. The general condition and course of the adaptation period of newborns born from pregnancies complicated by cervical cancer do not differ from the condition of newborns born in the appropriate weeks of pregnancy by healthy mothers [28,29].

In describing this case, we would like to highlight several aspects. (1) Aggressive, difficult to treat cancer additionally coexisting with pregnancy turned out to be a challenge for the entire therapeutic team. Previous literature reports on GCC of the cervix and oncological experience allowed us to determine the optimal treatment. (2) The hope and goal that the patient set for herself was the reason to overcome the ailments accompanying chemotherapy on the way to both health and motherhood. We often have to decide on the method of treatment. The possibility of various therapeutic options makes it extremely important to accurately present all the pros and cons of a given method. (3) Our patient was faced with many ethical dilemmas, because, on the one hand, her life was important, but also the desire to give birth to a healthy child. It is known that failure to treat pregnancy, especially in the case of such an aggressive and rare type of cancer, may be associated with disease progression. On the other hand, it is always accompanied by concerns about the impact of the applied therapy on the developing fetus. (4) From a legal point of view, taking any action or not taking any action requires the patient’s written informed consent each time.

## 4. Conclusions

Cervical GCC is a cancer that is not described in pregnant women. The history of our patient showed that treatment with chemotherapy in the second and third trimesters of pregnancy, and surgery and radiation therapy after childbirth, seem to be the optimal method of therapy for both the mother and her baby.

## Data Availability

The data presented in this study are available on request from the corresponding author.
